# Granuloma Caused by Carbon Deposition in the Dermis

**DOI:** 10.1155/2014/686489

**Published:** 2014-02-20

**Authors:** Rintaro Shibuya, Yuichiro Endo, Akihiro Fujisawa, Miki Tanioka, Yoshiki Miyachi

**Affiliations:** Department of Dermatology, Graduate School of Medicine, Kyoto University, 54 Shogoin, Kawahara-cho, Sakyo-ku, Kyoto 606-8507, Japan

## Abstract

Pencil core granuloma is characterized by a delayed foreign-body reaction against retained fragments of pencil lead. Previous case reports presented pencil core granuloma resembling malignant melanoma, haemangioma, or soft tissue sarcoma. We present a case of pencil core granuloma arising from the palm 25 years after the initial injury. The patient presented a bluish nodule that had been present over 25 years before. The nodule initially measured 5 mm in diameter. However, five years before presentation, it suddenly enlarged to the size of 30 mm during six months. Computed tomography (CT) of the lesion revealed a linear radiopaque structure of 8 mm long with a mass on its distal end. Surgical resection revealed a bluish muddy mass and pencil lead. Histological examination revealed degenerative tissue with calcification surrounded by massive amounts of black granular material in the middle and lower dermis.

Pencil core granuloma is characterized by a delayed foreign-body reaction against retained fragments of pencil lead. Previous case reports presented pencil core granuloma resembling malignant melanoma, haemangioma, or soft tissue sarcoma. We present a case of pencil core granuloma arising from the palm 25 years after the initial injury. The granuloma contained a pencil lead of 8 mm long without obvious breakdown.

A 67-year-old woman was referred to our department for evaluation of a bluish nodule on the palm of her right hand (Figure 1, arrow). The nodule had been present for 25 years, initially measuring 5 mm in diameter. However, five years before her presentation, it suddenly enlarged to the size of 30 mm during six months. Then, the tumor ceased its rapid growth and its size remained unchanged thereafter. The tumor was solid and hard, measuring 30 mm in diameter. The rest of the findings of her physical examination were unremarkable. Computed tomography (CT) of the lesion revealed a linear radiopaque structure of 8 mm long with a mass on its distal end (Figure 2, arrow). The total excision of the tumor was performed, revealing a bluish muddy mass that contained a piece of material that resembled pencil lead (Figure 3). Histological examination revealed degenerative tissue with calcification surrounded by massive amounts of black granular material in the middle and lower dermis. Histiocytes laden with fragments of black graphite were also observed at the peripheral of the degenerative tissue (Figure 4).

Pencil core granuloma is characterized by a delayed foreign-body reaction against retained fragments of pencil lead. In our case, the patient reported that she had stuck her hand with a pencil 25 years before and that a bluish nodule had been present since then. Five years ago, however, it suddenly started to enlarge to the size of 30 mm over six months and appeared similar to a nodular type of MM. Despite the past history that she had stuck her hand with a pencil, the lesion could not be distinguished from MM because MM may arise from various kinds of injuries. In our case, CT was useful since it showed a clear shape of the pencil lead [1].

Histopathological study of our case revealed a remarkable tissue necrosis and disintegration of pencil lead with scanty infiltration of giant cells or lymphocytes. However, some examinations reported that many giant cells and epithelioid cells were observed in pencil core granuloma [2, 3]. Hatano et al. attributed this difference to granulomatous reactions at different stages [4]. Although pencil lead generally induces a nonallergic granulomatous reaction, it might induce transient activation of macrophages during the rapid growth phase of the tumor. This implies that a high-turnover phase may precede the establishment of the quiescent lesion. In our case, the lesion was excited five years after the rapid growth phase, reflecting the scanty infiltration of giant cells or lymphocytes. Further evaluation is required for the detailed mechanism of activation of macrophages that triggers the rapid growth of the granuloma.

## Figures and Tables

**Figure 1:**
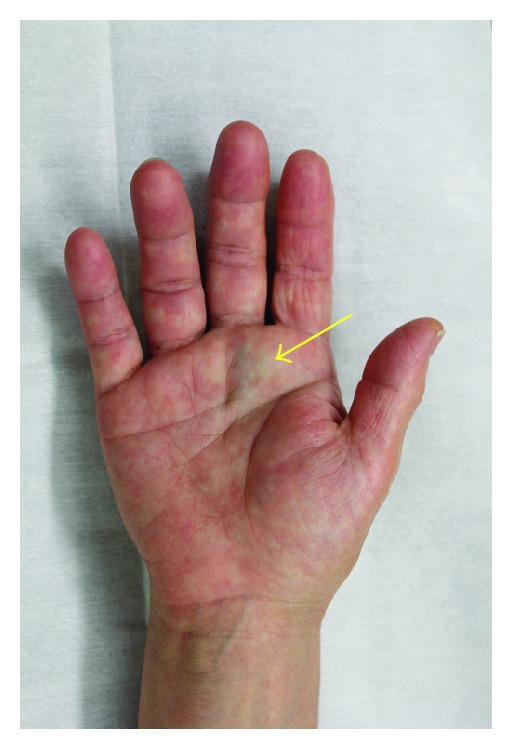
A bluish tumor of 30 mm in diameter was observed on the palm of her right hand.

**Figure 2:**
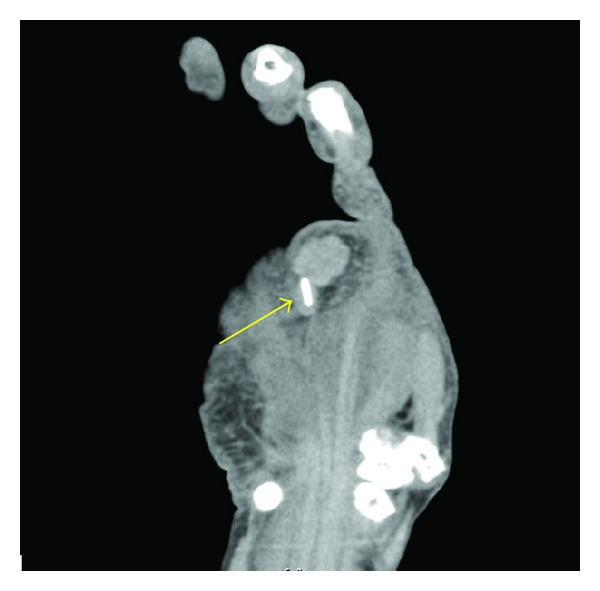
Contrast-enhanced CT showing a linear radiopaque structure of 8 mm long with a mass on its distal end.

**Figure 3:**
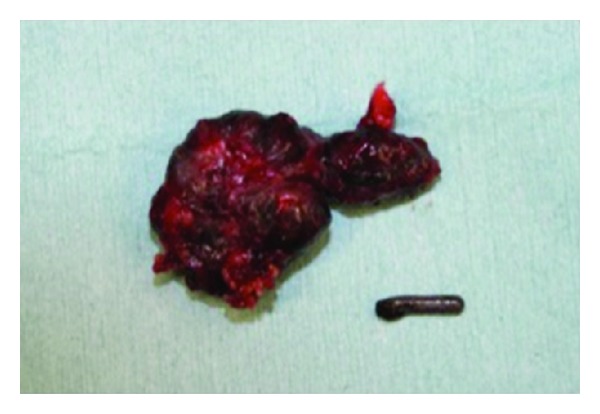
A black-pigmented fragment of 28 × 30 mm along with a lead was removed.

**Figure 4:**
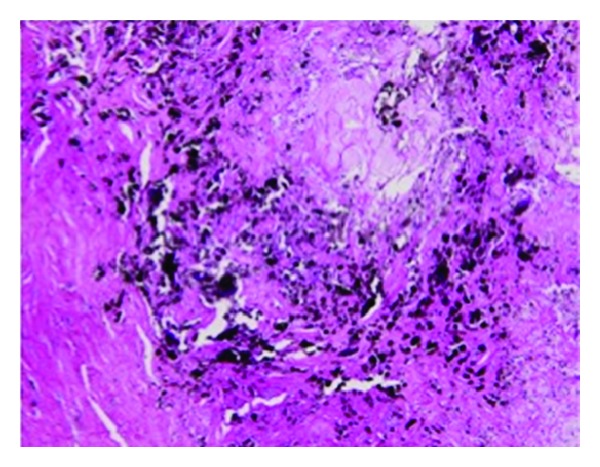
HE stain of the specimen revealed degenerative tissue with calcification and granulomas associated with histiocytes laden with fragments of black graphite (Hematoxylin eosin stain, ×100).
